# The Use of Iloprost in the Treatment of Bone Marrow Edema Syndrome of the Proximal Femur: A Review and Meta-Analysis

**DOI:** 10.3390/jpm12111757

**Published:** 2022-10-23

**Authors:** Timo Zippelius, Patrick Strube, Sebastian Rohe, Peter Schlattmann, Oliver Dobrindt, Thomas Caffard, Hassan Awan Malik, Chris Lindemann, Georg Matziolis, Sabrina Böhle

**Affiliations:** 1 Department of Orthopedic Surgery, University of Ulm, Oberer Eselsberg 45, 89081 Ulm, Germany; 2 Orthopedic Department, Jena University Hospital, Campus Eisenberg, 07607 Eisenberg, Germany; 3 Institute of Medical Statistics, Computer Sciences and Documentation, Jena University Hospital, 07743 Jena, Germany

**Keywords:** ilomedin, iloprost, prostaglandin, bone marrow edema syndrome, femoral head necrosis, osteonecrosis, meta-analysis, review

## Abstract

Objective: The aim of this meta-analysis was to investigate the impact of intravenous iloprost therapy on pain, function, edema changes, and follow-up surgery in bone marrow edema syndrome of the proximal femur. Methods: A systematic literature search up to May 2022 was performed to find relevant papers that made a statement about the outcome of intravenous iloprost therapy alone. Factors such as the Visual Analog Scale (VAS), Harris Hip Score (HHS), edema reduction, and follow-up interventions were considered. These were compared using Forest plots. Results: In 11 studies, 190 proximal femora with bone marrow edema syndrome that received intravenous iloprost therapy without further therapeutic intravenous or surgical intervention such as core decompression were studied. There was a significant mean improvement in VAS by 3.3 cm (2.07–4.5 cm) (*p* < 0.001) and HHS by 24.36 points (18.23–30.49) (*p* < 0.001) 3–6 months after receiving iloprost therapy. Only in 9.3% of cases (1.1–24.3%) did no clinical or radiological improvement occur. Conclusions: It could be shown that the existing publications support intravenous therapy with iloprost in patients with bone marrow edema syndrome and result in good clinical outcomes.

## 1. Introduction

Bone marrow edema (BME) is a disease of the bones that involves significant pain and functional limitations [1]. In some cases, it progresses to avascular necrosis (AVN) or osteonecrosis (ON), which leads to destruction of the affected joint, so that rapid recognition and adequate treatment are essential not only to achieve pain relief but also to preserve the joint [1,2]. Especially in the early stages of ON, differentiation from bone marrow edema is difficult or impossible [3,4,5,6]. Numerous studies have suggested that there is no single pathomechanism for the onset of bone marrow edema (BMES), but a multifactorial etiology [1,7,8]. Although the exact mechanism remains unclear, there is increased fluid accumulation in the interstitial marrow space which is triggered by local venous pressure increase. Multiple causes can lead to a BME: trauma-induced, degenerative, inflammatory, ischemic, infectious, metabolic, iatrogenic or neoplastic lesions [9]. Moreover, there is some controversy over whether BME is a separate disease or an early stage of an AVN [5,10,11] that leads to change of the local microcirculation [12,13,14]. In the conservative treatment of BMES, there is a wide range of therapeutic options and medications. The various treatment options include mechanical unloading and pain management, magnetic therapy and extracorporeal shock wave therapy, and hyperbaric oxygen. The best-studied methods include mechanical unloading with the administration of bisphosphonates as well as the prostaglandin derivative iloprost (ilomedin) [15]. Numerous studies have demonstrated the beneficial effects of intravenous iloprost therapy in the treatment of the initial stage of osteonecrosis with BMES in various joints [1,2,6,7,8,16,17,18,19,20,21]. It should be noted that intravenous iloprost administration is an “off-label use” and should only be performed under inpatient conditions with circulatory monitoring [22]. The synthetic iloprost has the pharmacodynamic profile of the endogenous prostanoid, epoprostenol (PGI2; prostacyclin), which is a universal and potent inhibitor of platelet activation. Iloprost displays some fibrinolytic activity, decreases neutrophil adhesion and chemotaxis, is an arterial vasodilator and decreases peripheral vascular resistance and mean arterial blood pressure. This could be because of an increase in smooth muscle cAMP secondary to receptor activation, but the reason is controversial [23]. Regarding the pharmacokinetics, iloprost is completely metabolized by β-oxidation with 70% renal excretion of the metabolites and 12 to 17% faecal excretion [23].

The aim of this work was to perform a meta-analysis of available studies on the treatment of BMES with iloprost to further elucidate the effects on pain, function, MR-tomographic edema changes, and surgery rate.

## 2. Methods

### 2.1. Literature Search

A systematic electronic literature search was conducted via PubMed (National Library of Medicine) until May 2022 by two independent persons to find relevant papers on the treatment of BME with iloprost. For this purpose, the terms “ilomedin”, “iloprost”, “prostaglandin”, “prostacyclin”, “femoral”, “hip”, “edema”, and “bone marrow edema” in various combinations were searched without time limit. A full PubMed search string was added online. Moreover, all literature lists of the included trial articles were screened by two reviewers.

### 2.2. Inclusion Criteria

Once studies were identified, they were further filtered using the following inclusion criteria before analysis:

- Intravenous iloprost therapy alone;
- Localization of the BME at the proximal femur;
- German or English language;
- Documentation of HHS, pain on a VAS, or MRI findings over a period of at least one month;
- If applicable, documentation of surgery rate and conversion to total joint arthroplasty.

Studies that examined multiple groups or localizations were included if conclusions could be explicitly drawn regarding intravenous iloprost therapy alone at the proximal femur. With regard to the PICOS criteria, we proceeded as follows: all participating patients were diagnosed with bone marrow edema at the proximal femur with the intervention intravenous iloprost therapy alone and the documented outcome. A control group was not necessary. Regarding the study design, we included prospective, retrospective studies, long-term pilot studies, and case reports.

### 2.3. Data-Extraction

After selecting studies based on the in-/exclusion criteria, two investigators independently conducted data extraction. Attention was paid to the pain on a Visual Analog Scale (VAS) and the Harris Hip Score (HHS) to assess the change in pain with iloprost therapy. In addition, the outcome after therapy was assessed in terms of improvement in pain, function, MR-tomographic changes, and follow-up surgery such as decompression or even conversion to total hip arthroplasty.

The following outcomes were extracted from the studies by two independent reviewers using Excel:

- VAS (before and after intervention to form a delta VAS);
- HHS (before and after intervention to form a delta HHS);
- Named as nonresponders or, if not named, failures in terms of subjective or/and MR-tomographic unchanged or worsened condition;
- Follow-up surgery including conversion to total joint arthroplasty.

### 2.4. Exclusion Criteria

Studies in which patients received additional surgical intervention such as core decompression or bisphosphonate therapy were excluded. Studies without documentation of VAS or HHS, as well as without recording of these scores before intervention, or without MR-tomographic evaluation, as well as studies without clear differentiation of results regarding the proximal femur were also excluded.

### 2.5. Risk of Bias Assessment

Two investigators independently assessed the quality of the studies involved. Risk of bias was assessed for each study using the Cochrane Risk of Bias Tool which included seven sources of bias, including randomization process, allocation concealment, blinding of participants and personnel, blinding of outcome assessment, incomplete outcome data, selective outcome reporting, and other potential bias [24]. Each study was examined based on the above seven aspects and subsequently assessed as at low risk of bias, high risk of bias, or unclear risk.

### 2.6. Meta Analysis

Studies were analyzed for statistical heterogeneity using an I^2^ test. In case of homogeneity with low I^2^, to obtain valid effect estimates, fixed-effects model was used for evaluation. When heterogeneity was demonstrated, the random-effects model was used. The results are presented graphically in Forest plots 

## 3. Results

### 3.1. Identification and Selection of Study Data and Study Characteristics

**Figure 1:**
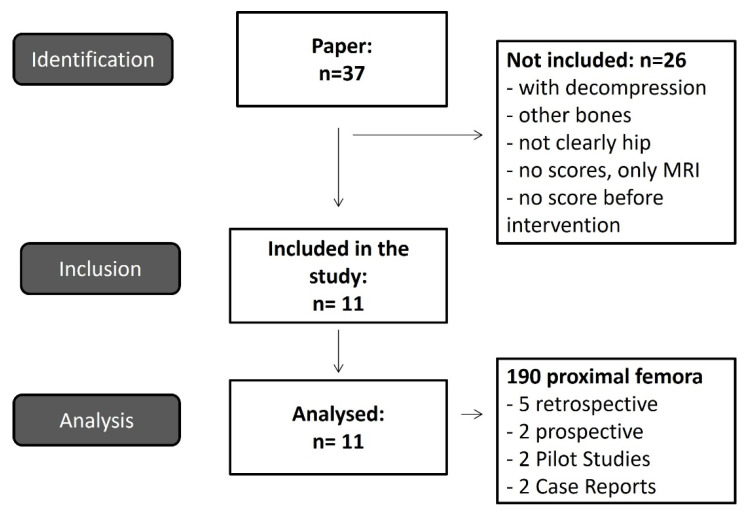
Flow Chart of inclusion of studies for the meta-analysis.

The online search using iloprost alone yielded more than 2900 papers. Combining the above keywords yielded 37 relevant clinical studies, including one dissertation that addressed iloprost therapy as part of prospective, retrospective study, long-term pilot studies, and case reports. Eleven studies met the inclusion criteria (Figure 1, Table 1) [2,6,18,25,26,27,28,29,30,31,32]. Twenty-six studies were excluded (see Table 2). These were predominantly retrospective studies. In addition, the 11 studies were published between 2004 and 2018 in German [32] or in English. A total of 190 proximal femora were studied because some patients had a diagnosis of femoral head necrosis bilaterally. On average, 31.5% of the patients were women and 68.5% were men, excluding the study by Jäger et al. 2008 as no exact allocation could be made for the 42 proximal femora affected in the 50 patients. The side effects mentioned in the studies with symptoms such as flushing, headache, or nausea were relatively mild, so only one patient had to discontinue the intervention in the 11 studies mentioned.

### 3.2. Risk of Bias

**Figure 2:**
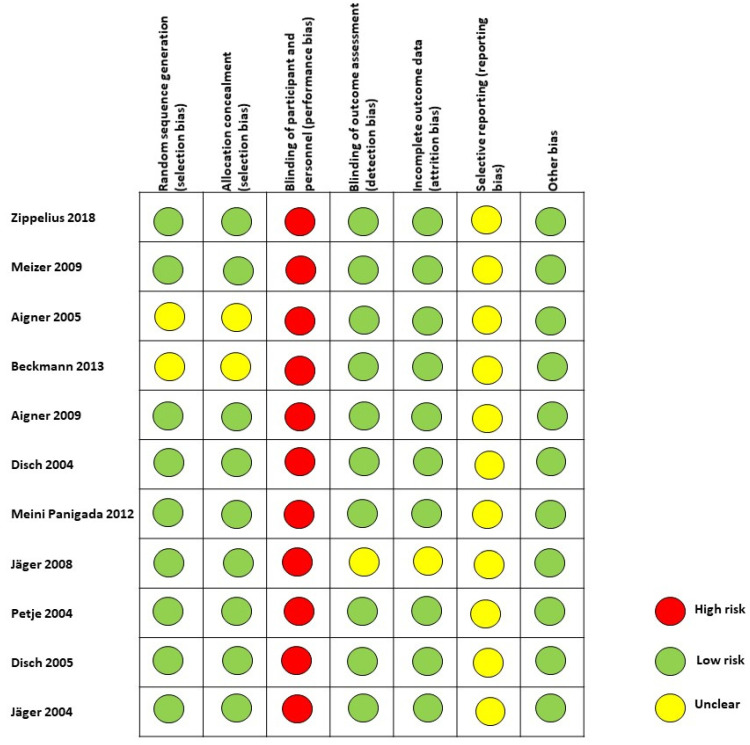
Risk of bias for each study. Legend: red, high risk; Yellow, unclear risk; green, low risk [2,6,18,25,26,27,28,29,30,31,32].

Figure 2 presents the summary of the risk of bias for each included study.

### 3.3. Heterogeneity of Included Studies

As a result of the heterogeneity tests, the study results were homogeneous with regard to conversion to total hip arthroplasty (I^2^ = 0%) and reoperations such as Salter´s osteotomy, femoral varization osteotomy, or core decompression (I^2^ = 16.1%). Measurements of improvement after iloprost therapy in terms of MR-tomographic edema reduction and subjective sensation as well as HHS and VAS before and after therapy showed significant heterogeneity. Therefore, 77.3–88.4% can be attributed to mathematical study heterogeneity rather than random sample variance. To account for these differences, a random-effects model was used to perform the calculation [53].

### 3.4. Therapeutic Effects after Intervention with Iloprost

**Figure 3:**
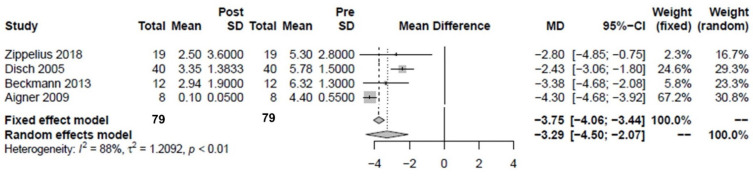
Forest plot showing the effect of iloprost therapy on the Visual Analog Scale (VAS) for pain. There was a significant difference (*p* < 0.01) [2,6,18,27].

For subjective assessment of pain, the VAS was determined in some studies before and after intravenous iloprost therapy (*n* = 7). Of these, three studies were excluded in which either multiple bones were examined and the VAS could not be related to the proximal femora alone [30,32] or in which the VAS was determined only after the second iloprost therapy [29]. The study regarding the improvement of pain by the intervention (delta VAS) showed a significant improvement of 3.3 cm (2.07–4.5 cm) (Figure 3). Except for the study by Zippelius et al., in which the VAS was only determined after approximately 29 months [2], the postintervention VAS comparison was made after three months. Some studies also determined further time points, which, however, did not produce any significant changes. The study by Aigner et al. was divided into pain at rest and pain on effort. Pain at rest was used [27]. Zippelius, Disch and Beckmann did not provide any information regarding the exact pain domain [2,6,18].

**Figure 4:**
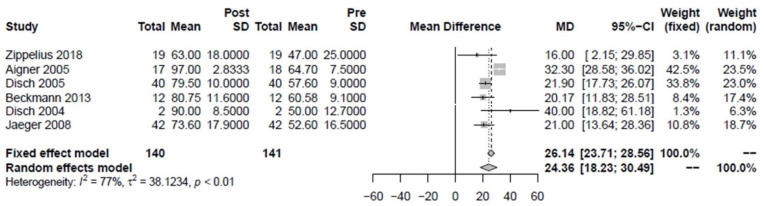
Forest plot showing the effect of iloprost therapy on Harris Hip Score (HHS). There was a significant difference (*p* < 0.01) [2,6,18,27,28,30].

In some studies (*n* = 7), the HHS was determined as a combination of subjective and objective assessment criteria with additional parameters such as function, function in everyday life, and physical examination, in addition to pain. Again, one study was excluded in which the HHS was determined only after the second therapy [29]. The HHS improved on average by 24.36 points (18.23–30.49) (Figure 4). For a more accurate influence in the meta-analysis, the time of HHS determination after three months could be used in most studies, as with the VAS. Again, the study of Zippelius et al. with the long-term influence of about 29 months [2] as well as the study of Disch et al. 2004 with one month follow-up differed [28].

**Figure 5:**
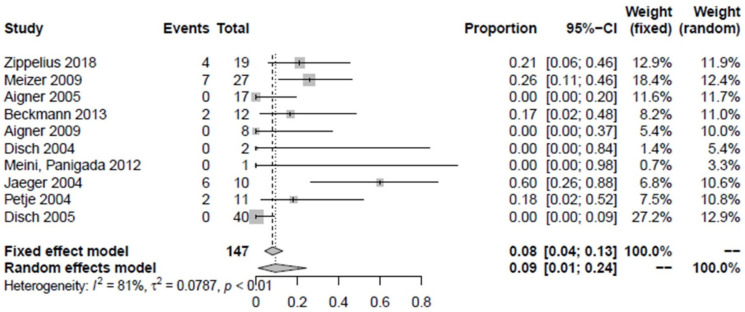
Forest plot showing the treatment failures after iloprost therapy for the treatment of bone marrow edema syndrome in terms of subjective pain relief and/or edema reduction on MRI. There was a significant difference (*p* < 0.01) [2,6,18,25,26,27,28,29,31,32].

**Figure 6:**
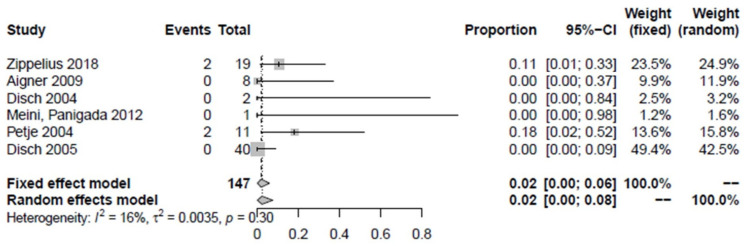
Forest plot showing hip joint preservation surgery after intravenous iloprost therapy [2,6,27,28,29,31].

**Figure 7:**
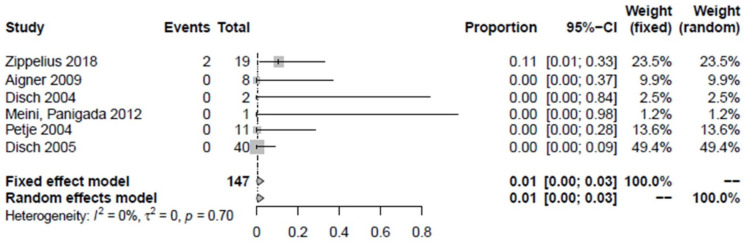
Forest plot showing the conversion to total hip arthroplasty [2,6,27,28,29,31].

Of the proximal femora examined as “non-responders”, 9.3% did not improve in terms of pain and/or MRI findings after the treatment (1.1–24.3%) (Figure 5). The study by Jäger et al. 2008 was not included in the determination of improvement because the proximal femora could not be precisely identified. However, it is known that 15 joints of stages ARCO III and IV were not affected [30]. Intravenous iloprost therapy improved the bone marrow edema syndrome in 90.7% (75.7–98.9%, *p* < 0.01). Overall, 1.9% of proximal femora underwent hip preservation surgery (0–5.9%) (Figure 6) which can be broken down into one Salter´s osteotomy, one femoral varization osteotomy, and two core decompression procedures. A total of 0.6% of all proximal femora previously treated with iloprost without concomitant surgical intervention were converted to total arthroplasty during follow-up (0–3.4%, *n* = 2) (Figure 7).

## 4. Discussion

The aim of the present meta-analysis was to investigate the influence of intravenous iloprost therapy on pain, function, and follow-up surgery in bone marrow edema syndrome of the proximal femur. The meta-analysis of the 11 included studies showed subjective relief of symptoms and/or edema reduction on MRI of bone marrow edema syndrome in 90.7% of cases with singular iloprost therapy (75.7–98.9%, *p* < 0.01). Twenty-one of 190 proximal femora did not respond to iloprost therapy, and six of these underwent surgical intervention after therapy (3.2%). The 2008 study by Jäger et al. was only included in the calculation of the HHS, but not in the further statistical analyses as no precise delineation could be performed here for all 117 bones studied [30]. Pain relief with iloprost therapy occurred in many cases during the five-day administration [6,28,29].

Nevertheless, the included studies show heterogeneity in some characteristics, which complicates the interpretation of the results. The nonresponders were identified differently in the individual studies: while in Zippelius et al. as well as Petje et al. these nonresponders were defined by MRI progression as well as follow-up surgery [2,31], the others in Meizer et al. and Beckmann et al. were directly labeled as nonresponders in the respective study [18,25]. It is important to emphasize here that Meizer et al. defined seven nonresponders by a nonresponse MR-tomographically [25], whereas Beckmann et al. no longer considered these proximal femora as nonresponders if the findings on MRI were unchanged but there was a clear pain response. Therefore, only the two nonresponders mentioned in the study were used in the present evaluation. However, it must be critically discussed whether the six MRI-tomographically unchanged or progressive proximal femora mentioned in the study should have been used for standardization [18]. Furthermore, in Jäger et al. 2004, nonresponse to therapy could be determined exclusively via MRI diagnostics, which, according to the VAS, affected all 20 bones examined (10 of which were proximal femora) [32].

Even though all studies were calculated with an iloprost therapy over 6 h for five days, the doses were not the same in all studies, since in some cases treatment involved a constant dose, a dose reduction in case of side effects, or an increased dose over the therapy period. In addition, the studies diverged with regard to the loading of the affected hip after therapy between full loading and partial loading for up to six weeks. Constant doses were administered in the following studies and can be divided into weight-independent and weight-adapted studies: Aigner et al. used 20 µg [26,27] and Meizer et al. 50 μg with a possible dose reduction to 20 μg in the event of side effects [25]. Weight-adapted constant doses were used by Zippelius et al. with 0.5 ng/kg [2], Petje et al. with 2 ng/kg [31] and Meini-Panigada et al. with 2 ng/kg and repeated after 4 weeks with 1.5 ng/kg [29]. A weight-adapted dose increase was recommended by Beckmann et al. with day 1 20 μg/kg, day 2 30 μg/kg, day 3–5 40 μg/kg by Beckmann et al. [18] and with day 1 0.5 μg, day 2 0.75 μg, day 3–5 1 μg/kg by Jäger et al. and Disch et al. [6,28,30]. One dosage remains unknown [32]. Frequently, no information was provided regarding postinterventional mobilization. To obtain valid effect estimates, the random-effects model was used with regard to improvement after iloprost therapy as well as HHS and VAS before and after therapy. In Meini et al., a case report was made of a patient who received iloprost therapy again after four weeks. Edema reduction and relief of symptoms were already achieved after the first therapy. However, because only a short-term statement of a singular iloprost therapy could be made, VAS and HHS were not included in our statistical analysis [29]. In addition, we cannot exclude the possibility that co-authorships used the same patient population to generate the study. Likewise, the studies measured different follow-up periods (3–32 months), so possible further follow-up surgeries as well as conversions to total hip arthroplasty could have occurred.

A limitation of the meta-analysis is the small number of patients; higher patient numbers and further long-term results are required for definitive conclusions. There was no a priori protocol. In addition, the different patient populations with femoral head necrosis from I to IV according to the Association Research Circulation Osseous (ARCO) staging is a fundamental limitation, as the 2015 S3 guideline recommends iloprost therapy only for stage I and II [19]. Therefore, some studies such as Zippelius et al. excluded ARCO III and IV [8]. Jäger et al., for example, also investigated therapy in ARCO stages III (*n* = 9) and IV (*n* = 1), among others [30]. However, in the study by Beckmann et al., it was interesting to note that one of two nonresponders with a hip with ON (ARCO II) who failed to improve at an early stage had been successfully treated with iloprost six months earlier due to contralateral BME [18]. With monotherapy with iloprost as well as with decompression of ON, the results were not as promising as with BME [18]. Nevertheless, iloprost was shown to be an option to achieve pain relief and mobilization, even at higher ARCO stages. Here, however, iloprost therapy should be seen as part of a conservative treatment regimen and supplemented by other measures if necessary [6].

In summary, there is a discrete improvement in bone marrow edema syndrome with intravenous iloprost therapy alone. In most studies, this effect occurs within the first days or weeks. According to Aigner et al., a comparison of core decompression with singular iloprost therapy showed equally good results (*n* = 20 vs. *n* = 18) [26]. According to Beckmann et al., the combination of intravenous iloprost and retrograde tapping seems to be the most promising treatment method compared with iloprost therapy or tapping alone (*n* = 12 per group). Although improvement in HHS, WOMAC score, SF-36 score, and VAS was shown three months and one year after therapeutic intervention, the greatest effects were obtained with the combination [18]. Thus, it can be concluded that intravenous iloprost therapy has a positive effect on bone marrow edema syndrome in the proximal femur, but may not always be accompanied by edema reduction on MRI, improvement in hip joint function, and/or pain reduction, especially in later ARCO stages.

This study has limitations. Two reviewers using the protocol for inclusion and exclusion criteria thoroughly examined the studies and carefully identified specific study designs, biases within studies, differences between studies, and reporting biases. However, it must be said that the literature that accurately address the stated topic and allows for analysis is limited, as evidenced by the number of studies included.

## 5. Conclusions

Intravenous iloprost therapy has an impact on bone marrow edema syndrome in the proximal femur. It can produce a reduction in pain, improvement in function, and MR-tomographic reduction in the extent of edema.

## Figures and Tables

**Table 1:** Literature analysis of the included studies.

Author	Year	Study Design	Number of Proximal Femora	Gender	Dose	MRI Result	Failure	Control Group	Control after	Follow-Up	Partial Load	Besonderheit	VAS	HHS
Zippelius [2]	2018	retrospective	19	7 women, 12 men	0.5 ng/kg/min over 6 h over 5 days	After 3 months: 15/19 complete edema regression	4 surgeries (2× cannulation, 2× TEP)	/	3, approx. 29 months	29 ± 11 months	6 weeks		X only after 29 months	X only after 29 months
Meizer [25]	2009	retrospective	27	8 women, 19 men	50–20 μg over 6 h for 5 days; start with 50, reduce if needed	After 4 months: 20/27 improved on MRI, unchanged, 3 worsened	7 nonresponders (named)	/	4 months	4 months	3 weeks	81% less pain at rest, 63% less activity pain	Other score	-
Aigner [26]	2005	retrospective	18	4 women, 13 men	20 μg over 6 h for 5 days	After 3 months: Complete edema regression in all femora	/	Core decompression group	3, 12 months	11 months	Partial (for 5 patients for 3 weeks)	In the iloprost group, one patient had to discontinue treatment due to severe headache (n post: 17 femora)	-	X after 3 months
Beckmann [18]	2013	retrospective	12	3 women, 9 men	20–40 μg over 6 h for 5 days, day 1 20 μg, day 2 30 μg, day 3–5 40 μg	After 3 months: Reduction from BME, not from ON	2 nonresponders by pain indication (named)	Core decompression, core decompression with ilomedin	3, 12 months	13 months (11–16) (all groups)	6 weeks	The combination of iloprost and tapping shows the best results	X after 3 months	X after 3 months
Aigner [27]	2009	prospective	8	6 women	20 μg over 6 h for 5 days, start 10 days after birth	After 3 months: Complete remission in 5, subtotal in 1 pat (all BME.) No progression at last follow-up	/	/	1, 3 months	31 months (14–43)	unknown	All six subjects improved immediately during the first 2 weeks after initiation of intravenous therapy	X after 3 months at rest	-
Disch [28]	2004	Case Report	2	1 man (with sickle cell anemia)	0.5–1.0 μg/kg/min over 6 h/d for 5 days; day 1 0.5 μg, day 2 0.75 μg, day 3–5 1 μg	After 3 months: Significant reduction of edema in both femora	/	/	1, 3 months	3 months	unknown	Already after the 3rd day less pain. Further improvement in the next 4 weeks regarding ROM	-	X after 1 month
Meini, Panigada [29]	2012	Case Report	1	1 woman	2 ng/kg/min over 6 h for 5 days, after 4 weeks repeat with 1.5 ng/kg/min over 6 h for 5 days	After 4 weeks, reduction of edema, then iloprost again. Then, after 4 weeks, further reduction; after three more months, control/complete remission	/	/	1, 2, 5 months after first treatment	5 months after first treatment	unknown	During treatment with iloprost from the third day significant reduction of pain and joint dysfunction	X after second therapy	X after second therapy
Jäger [30] ^1^	2008	prospective	42	28 women, 22 men (total 117 bones)	0.5–1.0 ng/kg/min over 6 h for 5 days	Significant reduction of edema after 3 and 6 months. After 6 months, complete regression in 65 of 117 bones. Advanced ARCO stages (III, IV) were not affected by iloprost	Not exactly named. However, ARCO III and IV unchanged (total 15 joints)	/	5 days, 3, 6 months		unknown		X VAS in all bones	X after 3 months
Petje [31]	2004	Long-term pilot study	11	3 women, 4 men	2 ng/kg/min over 6 h for 5 days	ON progression in 2 patients	2 Perthes children with Salter surgery and femoral varization osteotomy	/	/	32 months (12–48) all bones (45 patients)	unknown		-	-
Disch [6]	2005	prospective, case-controlled	40	7 women, 26 men	0.5–1.0 μg/kg/min over 6 h/d for 5 days; day 1 0.5 μg, day 2 0.75, day 3–5 1 μg	Edema reduction in all patients after 3 months	/		1,3, and approx. 25 months	25 months (11–37)	unknown	Group I: 16 isolated BME (ARCO I), Group II: 17 ON (ARCO II, III)	X after 3 months	X after 3 months
Jäger [32]	2004	prospective	10	2 women, 5 men (total 20 bones)	Unknown dose for 5 days	After 3 months, reduction in 4 proximal femora, no change in another 4, and in 2 (the latter already previously ARCO III & IV)	6 after MRI		3 months	3 months	unknown	unknown	Yes, but all joints at VAS	-

^1^ In this study, 50 patients with 117 affected bones were examined. Of these, 42 bones were proximal femora. A precise assignment of sexes to femora was not made.

**Table 2:** Excluded studies.

Author	Year	Reason for Exclusion
Aigner [33]	2001	Location (talus)
Aigner [34]	2002	Location (forefoot)
Aigner [3]	2002	Location (acetabulum)
Aigner [35]	2003	Location (hindfoot)
Aigner [36]	2005	Location (foot)
Aigner [37]	2008	Location (knee)
Anagnostakos [38]	2013	No clear differentiation of results regarding the proximal femur (21 bones)
Arazi [39]	2006	Location (ankle)
Arazi [40]	2011	Location (os metatarsale)
Baier [41]	2013	Location (knee, foot)
Claßen [20]	2016	No clear differentiation of results regarding the proximal femur (136 bones)
Hörterer [42]	2018	Location (foot, ankle)
Huang [43]	2020	No treatment with iloprost
Jäger [44]	2009	3 out of 8 patients had a core decompression, no clear differentiation
Jäger [17]	2011	No clear differentiation of results regarding the proximal femur (20 bones)
Lackner [45]	2005	Observational Study: 3 out of 9 patients with iloprost without a clear differentiation
Mahmoudi (dissertation) [46]	2009	Most likely the same cohort as Jäger 2008, co-author
Mayerhoefer [47]	2007	Location (knee)
Mayerhoefer [48]	2008	Location (knee)
Meizer [12]	2005	No clear differentiation of results regarding the proximal femur (104 bones)
Petje [49]	2002	No scores, no clear differentiation
Pilge [50]	2016	Ilomedin therapy with core decompression and bone marrow aspirate
Röhner [7]	2014	Location (foot, ankle)
Tillmann [51]	2007	Location (knee, foot)
Tosun [52]	2020	No clear differentiation of results regarding the proximal femur (23 bones)
Zippelius [8]	2018	Location (knee)
